# Can We Optimize Antibiotic Use in Norwegian Neonates? A Prospective Comparison Between a University Hospital and a District Hospital

**DOI:** 10.3389/fped.2019.00440

**Published:** 2019-10-24

**Authors:** Christian Magnus Thaulow, Dag Berild, Hege Salvesen Blix, Anne Karin Brigtsen, Tor Åge Myklebust, Beate Horsberg Eriksen

**Affiliations:** ^1^Clinical Institute II, University of Bergen, Bergen, Norway; ^2^Department of Pediatrics, Haukeland University Hospital, Bergen, Norway; ^3^Institute of Clinical Medicine, University of Oslo, Oslo, Norway; ^4^Institute of Pharmacology, University of Oslo, Oslo, Norway; ^5^Department of Infectious Diseases, Oslo University Hospital, Oslo, Norway; ^6^Department of Drug Statistics, Norwegian Institute of Public Health, Oslo, Norway; ^7^Department of Pediatrics, Oslo University Hospital, Oslo, Norway; ^8^Department of Research and Innovation, Møre and Romsdal Hospital Trust, Alesund, Norway; ^9^Department of Pediatrics, Møre and Romsdal Hospital Trust, Oslo, Norway

**Keywords:** neonatal antibiotic use, antimicrobial resistance, pediatric antibiotic stewardship, antibiotic doses, antibiotic prescriptions

## Abstract

**Background:** Worldwide, a large proportion of neonates are prescribed antibiotics without having infections leading to increased antimicrobial resistance, disturbance of the evolving microbiota, and increasing the risk of various chronical diseases. Comparing practice between different hospitals/settings is important in order to optimize antibiotic stewardship.

**Aim:** To investigate and compare the potential for improved antibiotic stewardship in neonates in two Norwegian hospitals with different academic culture, with emphasis on antibiotic exposure in unconfirmed infections, treatment length/doses, CRP values and the use of broad-spectrum antibiotics (BSA). All types of infections were investigated, but the main focus was on early-onset sepsis (EOS).

**Methods:** We conducted a prospective observational cohort study of antibiotic use in a Norwegian university hospital (UH) and a district hospital (DH), 2017. Unconfirmed infections were defined as culture negative infections that neither fulfilled the criteria for clinical infection (clinical symptoms, maximum CRP >30 mg/L, and treatment for at least 5 days).

**Results:** Ninety-five neonates at the DH and 89 neonates at the UH treated with systemic antibiotics were included in the study. In total, 685 prescriptions (daily doses) of antibiotics were given at the DH and 903 at the UH. Among term and premature infants (≥ 28 weeks), 82% (75% at the UH and 86% at the DH, *p* = 0.172) of the treatments for suspected EOS were for unconfirmed infections, and average treatment length in unconfirmed infections was 3.1 days (both hospitals). Median dose for aminoglycoside was higher for term infants at the UH (5.96, 95% CI 5.02–6.89) compared to the DH (4.98, 95% CI 4.82–5.14; *p* < 0.001). At the UH, all prescriptions with aminoglycosides were gentamicin, while tobramycin accounted for 93% of all prescriptions with aminoglycosides at the DH.

**Conclusion:** There is a potential for reduction in both antibiotic exposure and treatment length in these two neonatal units, and a systematic risk/observational algorithm of sepsis should be considered in both hospitals. We revealed no major differences between the UH and DH, but doses and choice of aminoglycosides varied significantly.

## Introduction

Unnecessary use of antibiotics leads to increased rates of antimicrobial resistance (AMR) and is one of the main challenges in global health (1, 2). AMR rates are low in Norway compared to other countries but has increased during the last decade (3). The Norwegian government has introduced a National Strategy aiming for a 30% reduction in total antibiotic use, and a 30% reduction in the use of broad-spectrum antibiotics (BSA) in hospitals, by year 2020 (4).

Neonates and small children are particularly vulnerable to antibiotic exposure as the diversity of the gut microbiota increases and evolves during the first years of life (5). In addition to increased resistance rates (6, 7), early life antibiotic exposure is associated with the evolvement of various chronic diseases (8–10).

Worldwide, neonates with suspected sepsis are exposed to antibiotics, although only a small proportion have a confirmed infection (11–13). The interpretation of risk factors, clinical symptoms, and biomarkers is challenging, and may explain why antibiotic exposure rates in neonates vary between hospitals, also within the same countries (11, 14). A registry-based population study from Norway (2009–2011) showed that half of term-infants receiving antibiotics were not proven to have a bacterial infection (11). Use of BSA in Norwegian neonates is lower than in older children, but empirical choices of antibiotics vary, and there is a lack of evidence on neonatal dose regimes (15, 16).

Fifteen of the 68 hospitals registered in the database of The Norwegian Institute of Public Health hold a neonatal unit; seven of these units are situated in university hospitals while the rest are situated in smaller district hospitals (all are public hospitals). The university hospitals hold many academic positions and are expected to be in the frontline of developing clinical practice. We therefor speculate whether there are any clearly differences in antibiotic use between centrally located university hospitals and more rural located district hospitals.

The aim of this study was to explore antibiotic use among neonates with and without confirmed infection, with emphasis on choice and dosing of antibiotics, treatment duration, CRP values and the use of BSA. Furthermore, we assessed whether pattern of antibiotic use in neonates differs between university and district hospitals.

## Methods

### Setting and Design

We designed a prospective observational cohort study, collecting data from 2017 to describe and compare antibiotic use in neonates in a Norwegian university hospital (UH) (Oslo University Hospital, Ullevål) and a district hospital (DH) (Ålesund Hospital).

### Hospitals

The study population consisted of all neonates admitted to the neonatal units at the UH and the DH in the study periods. The DH has a neonatal intensive care unit (NICU) consisting of 13 beds and provides regional neonatal service for neonates from gestational age (GA) 26 weeks (after centralization of infants below 26 weeks to a regional UH from February 2017) and offers all kinds of intensive care apart from neonatal surgery and ECMO. The UH has a NICU consisting of 27 beds and provides regional service for neonates with all GA ages and all intensive care needs apart from ECMO and thoracic/heart surgery. Both hospitals mainly treat neonates admitted from the maternity ward, but at the DH critically ill infants (<3 month) can in certain circumstances be referred to the neonatal unit from home.

There are no official national guidelines for antibiotic treatments in neonates in Norway, and most hospitals have local guidelines. In 2017, both study hospitals recommended the use of an aminoglycoside in combination with ampicillin for the treatment of early-onset sepsis. For term infants, the UH recommended aminoglycoside to be dosed 6 mg/kg as one daily administration, while the DH recommended 5 mg/kg. Both hospitals recommended ampicillin to be dosed 50 mg/kg two times a day. None of the hospitals used specific algorithms/observations routines for deciding whether to start antibiotic therapy once neonatal sepsis was suspected. The communication between the laboratory and the neonatal departments is well-established in both hospitals, and both results from CRP analyses and blood cultures are easy and rapidly available for the treating clinicians. In both hospitals, positive blood cultures are alerted directly from the microbiologist in terms of a personal call to the on-duty physician.

### Data Collection

At the DH, data were collected from 1st of January−31st of December, 2017. The collection was performed by trained nurses working at the unit and double-checked by the project manager. At the UH, data were collected during 15 weeks in 2017; from 27th of March−20th of May and from 01st of November−31th of December. Data were collected by two MD‘s working at the unit and the quality control was performed by the project manager. Educational classes for data collectors were held before the start of the registration.

In both hospitals, patients receiving antibiotics were identified at 08.00 a.m. by the collectors every morning by evaluating all inpatients. In these neonatal wards, outpatient treatment of infections is very uncommon, thus we only included inpatients.

For registrations we modified and extended an international standardized point prevalence protocol developed by the European Center for Disease Prevention and Control (ECDC) (17). The data were stored in an electronical database.

Data collection included the total number of patients in the wards, the total number of patients receiving antibiotics, gender, GA at birth, birthweight, delivery mode, age and weight at the start of antibiotic treatment, type and dose (including intervals) of antibiotics, route of administration, treatment duration (in days), whether it was for treatment of infection or prophylaxis, indication for treatment/prophylaxis, respiratory support (any kind), maximum CRP value and results from blood cultures. For patients receiving antibiotics at the start or end of the registration period we obtained information from previous/remaining days of antibiotic treatment.

The total numbers of live births in the uptake area for both hospitals were collected from the maternity ward and also controlled with the Norwegian birth registry.

### Variables and Definitions

Term-infants were defined as GA ≥ 37 weeks, premature infants as GA 28–36 weeks and extremely premature infants as GA 23–27 weeks. All prescribed antibiotics were included in our analyses and described in relation to prescriptions, administrations, courses, and admitted patients. One prescription was defined as a daily dose with one antibiotic, an administration was defined as one single dose with one antibiotic, and a course was defined as antibiotic therapy/prophylaxis with one or more antibiotics for a certain indication in a certain continuous time range. Each patient registered at the wards during the daily registration was regarded as one bed day. Doses in mg/kg were based on birthweight until a higher body weight was recorded, and we only compared doses in term infants. Treatment duration was defined as number of days with antibiotic exposure. The total number of live births was used as a denominator for expressing antibiotic exposure within the first 3 days of life. Antibiotics were defined as antibacterials for systemic use (J01). Broad-spectrum antibiotics were defined as second- and third-generation cephalosporins, carbapenems, piperacillin/tazobactam and quinolones, according to the National Strategy against AMR (4).

Surgical prophylaxis was defined as antibiotics given immediately before, during or shortly after surgery to prevent infection. Medical prophylaxis was defined as antibiotics prescribed to prevent infection in patients at risk, but without infectious symptoms and without obtainment of blood culture. Cases where symptoms could be explained by infections, but also by other conditions (for instance prematurity, asphyxia) were not regarded as prophylaxis. Early-onset sepsis (EOS) was defined as suspected sepsis within the first 3 days of life and late-onset sepsis (LOS) when sepsis was suspected after 3 days of life. Other indications were only used if organ specific symptoms were present (such as skin infections) without suspected sepsis. In theory, all infant with clinical symptoms and exposure for blood culture were classified as sepsis treatments.

Treatments for suspected sepsis were divided in three categories: Culture positive sepsis (which required a positive blood culture and clinical symptoms), culture negative sepsis and no sepsis. The first two categories were regarded as confirmed infections. According to recommendations from the Norwegian Neonatal Network, the diagnosis of a culture negative neonatal sepsis (International Classification of Diseases, 11th revision, P36.9), should only be used if certain criteria are fulfilled; clinical symptoms, CRP >30 mg/L, at least 5 days of antibiotic therapy (or death before 5 days) and whenever other medical conditions are ruled out (18). Thus, we only included neonates with CRP >30 and with at least 5 days of antibiotic treatment (or death before 5 days) when defining culture negative neonatal sepsis. According to the same recommendations, growth of coagulase-negative Staphylococci in blood culture were only considered as neonatal sepsis with CRP >10 and at least 5 days of antibacterial therapy (or death before 5 days). The same method was used to measure CRP in both hospitals: Particle enhanced immune turbidimetry.

### Statistics

Statistical analyses were performed using SPSS Statistics 23 (SPSS Inc., Chicago, IL, USA). Comparisons of proportions were done using standard chi-square tests. Means and medians were compared using independent samples *t*-test and Moods median test, respectively. 95% confidence intervals of means were calculated assuming normal distribution, whereas confidence intervals of medians were calculated using the binomial distribution. Correlation was estimated using Pearson correlation coefficient. *P*-values <0.05 were considered significant. Because the DH discontinued their service for extremely premature infants with GA <26 weeks during the study period, antibiotic use in extremely premature infants (GA <28 week) was described without comparisons.

### Ethics

The study was approved by the Regional Committee for Medical and Health Research Ethics (2017/30/REK Midt) and by the Local Data Protection Officials at the two hospitals.

## Results

### Demographics and Characteristics

In total, 184 patients received 207 courses and 1,588 prescriptions of antibiotics. See Table 1 for comparisons of demographics and characteristics between the hospitals.

**Table 1:** Characteristics of neonates receiving antibiotics in two different Norwegian neonatal units in 2017.

	**Total**	**University Hospital**	**District Hospital**	***p*-value^**a**^**
**ALL**				
Patients, *n*	593	235	358	
Patients exposed to antibiotics, *n* (%)	184	89 (38)	95 (27)	n/a
Courses with antibiotics, *n*	208	108	100	
Prescriptions with antibiotics, *n*	1,588	903	685	
Bed days with antibiotics/total bed days (%, 95% CI)	856/5,486 (16)	492/2,714 (18, 17–19)	364/2,772 (13, 12–14)	n/a
Antibiotic exposure first 3 days/number of live births (%, 95% CI)	150/4,772 (3.1, 2.6–3.6)	73/2,091 (3.5, 2.7–4.3)	77/2,681 (2.9, 2.3–3.5)	n/a
**TERM INFANTS**				
Patients on antibiotics, *n* (%)	106 (58)	39 (44)	67 (71)	n/a
Courses with antibiotics, *n* (%)	108 (52)	40 (37)	68 (69)	n/a
Prescriptions with antibiotics, *n* (%)	769 (48)	301 (33)	468 (68)	n/a
Prophylaxis/treatments, % of courses	4.6/95.4	7.5/92.5	2.9/97.1	0.278
Male/Female, % of patients	67/33	72/28	64/36	0.401
Cecirian delivery/vaginal delivery, % of patients	25/72	31/62	22/78	0.228
GA (weeks), mean (SD)	39.8 (1.7)	40.1 (2.0)	40.0 (1.6)	0.821
Weigh at start of treatment (g), mean (SD)	3,798 (616)	3,823 (615)	3,774 (616)	0.701
Antibiotic exposure first 3 days/number of live births (%, 95% CI)	92/4,470 (2.1, 1.7–2.5)	36/1,967 (1.8, 1.2–2.4)	56/2,503 (2.2, 1.6–2.8)	0.346
**PREMATURE INFANTS**				
Patients on antibiotics, *n* (%)	40 (22)	16 (18)	24 (25)	n/a
Courses with antibiotics, *n* (%)	42 (20)	17 (16)	25 (25)	n/a
Prescriptions with antibiotics, *n*	281 (18)	127 (14)	154 (22)	n/a
Prophylaxis/treatments, % of courses	10/90	5.8/94.2	8.0/92.0	0.670
Male/Female, % of patients	58/42	38/62	71/29	0.041
Cecirian delivery/vaginal delivery, % of patients	65/30	50/38	75/25	0.253
GA (weeks), mean (SD)	32.1 (2.4)	31.5 (2.3)	32.7 (2.6)	0.172
Weigh at start of treatment (g), mean (SD)	1,872 (747)	1,481 (537)	2,115 (758)	*0.004*
Antibiotic exposure first 3 days / number of live births (%, 95% CI)	32/269 (12, 8–16)	13/95 (14, 7–21)	19/174 (11, 6–16)	0.471
**EXTREMELY PREMATURE INFANTS** ^**b**^				
Patients on antibiotics, *n* (%)	38 (21)	34 (38)	4 (4)	
Courses with antibiotics, *n* (%)	58 (28)	51 (47)	7 (7)	
Prescriptions with antibiotics, *n* (%)	538 (34)	475 (53)	63 (9)	
Antibiotic exposure first 3 days / number of live births (%)	26/33 (79)	24/29 (83)	2/4 (50)	n/a

a *A chi square test was used for proportions and Student's t-test for means. N/A means that statistic testing was not appropriate because of case mix differences between the hospitals*.

b *The DH only treated infants with GA <28 weeks between 1th of January and 15th of February.*

*• GA, Gestational age.*

*• Term infants (≥ 37 weeks), premature infants (28–36 weeks), extremely premature infants (23–27 weeks).*

*• Missing data: Delivery mode on three patients (GA>37 weeks) and two patients (GA 28–37 weeks) at the University hospital, weight at one patient (GA >37 weeks) and two patients (28–37 weeks) at the University hospital.*

### Antibiotic Prescriptions

**Figure 1:**
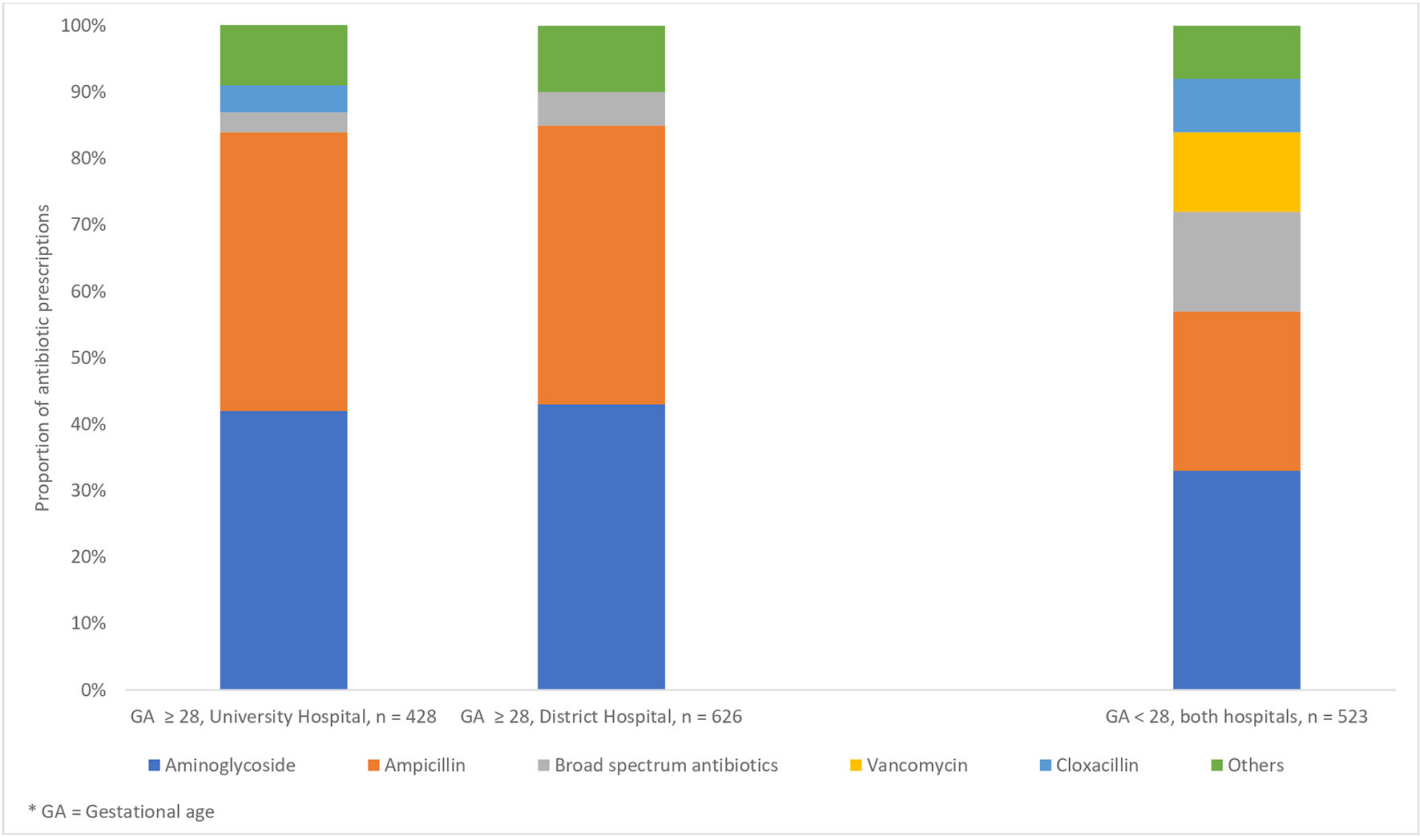
Distribution of antibiotic prescriptions in two Norwegian neonatal units. Broad- spectrum antibiotics are defined as second- and third generation cephalosporins and carbapenems.

For term and premature infants, aminoglycosides, and ampicillin accounted for the majority of antibiotic prescriptions, namely 84% at the UH and 85% at the DH (See Figure 1). Use of BSA was low (4.3% in total for both hospitals), but the proportion of prescriptions was significantly higher at the DH vs. the UH; 34 prescriptions (5.5%) vs. 11 prescriptions (2.6%), respectively (*p* = 0.023), see Table 2 for more information. Out of seven BSA courses prescribed at the DH, three were given for LOS, two for lower respiratory tract infection, one for EOS and one for infection in the CNS. Three of these patients received respirator treatment, and three were premature infants. The one course of BSA that was prescribed at the UH were given for EOS to a term infant receiving respirator treatment.

**Table 2:** Distribution of antibiotic exposure in two Norwegian neonatal units (GA >28) based on start of antibiotic exposure, 2017.

	**All**	**University Hospital**	**District Hospital**	**P-value^**a**^**
**ALL**				
Courses, *n*	150	57	93	
Prescriptions, *n*	1,050	428	622	
BSA^b^ prescriptions, n (%)	45 (4.3)	11 (2.6)	34 (5.5)	*0.023*
Courses including BSA, *n* (%)	8 (5.3)	1 (1.8)	7 (7.5)	0.128
**0–3 DAYS**				
Courses^c^, *n* (%)	124 (83)	49 (86)	75 (81)	0.405
Prescriptions, n (%)	861 (82)	369 (86)	492 (79)	*0.003*
BSA prescriptions, *n* (%)	15 (1.7)	11 (3.0)	4 (0.8)	*0.019*
Courses including BSA, *n* (%)	2 (1.6)	1 (2.0)	1 (1.3)	0.761
**3–10 DAYS**				
Courses^d^, *n* (%)	14 (9)	6 (11)	8 (9)	0.695
Prescriptions, n (%)	113 (11)	51 (12)	62 (10)	0.317
BSA prescriptions, *n* (%)	10 (9)	0 (0)	10 (16)	*0.002*
Courses including BSA, *n* (%)	2 (14)	0 (0)	2 (25)	0.202
**>10 DAYS**				
Courses^e^, *n* (%)	12 (8.0)	2 (3.5)	10 (11)	0.114
Prescriptions, n (%)	76 (7.2)	8 (1.9)	68 (11)	*<0.001*
BSA prescriptions, *n* (%)	20 (26)	0 (0)	20 (29)	0.102
Courses including BSA, *n* (%)	4 (33)	0 (0)	4 (40)	0.294

a *Chi square test.*

b *BSA: Broad-spectrum antibiotics are defined as second-and third generation cephalosporins and carbapenems.*

c *University hospital (UH) treatment: early onset sepsis (48), UH prophylaxis: maternal syphilis (1).*

*District hospital (DH) treatment: Early onset sepsis (73), DH prophylaxis: central catheter line (1), vesicourethral reflux (1).*

d *UH treatment: late onset sepsis (3), eye-infection (1). UH prophylaxis; vesicourethral reflux (2).*

*DH treatment: infection in skin, joint and bone (4), late-onset sepsis (3). DH prophylaxis: surgery of transposition of the great vessels (1).*

e *UH prophylaxis: tracheostomia (1), unknown (1).*

*DH treatment: Late onset sepsis (4), lower respiratory tract infection (3), infection in bone, joint and skin (2), CNS infection (1)*.

For extremely premature infants, aminoglycosides and ampicillin accounted for 57% of the prescriptions (both hospitals), and BSA use accounted for 15%. For all neonates, 119 (96%) of in total 124 prescriptions with BSA were second or third generation cephalosporins and 5 (4%) were carbapenems. At the UH, all prescriptions with aminoglycosides were gentamicin, while tobramycin accounted for 93% of all prescriptions with aminoglycosides at the DH.

### Startup of Antibiotics

Hundred and twenty-four (83%) out of 150 courses of antibiotics for term and premature infants were started during the first 3 days of life in both hospitals; day one (107, 71%), day two (10, 7%), day three (7, 5%). Table 2 shows that prescription rate in relation to starting time of the course varied significantly between the hospitals. For extremely premature infants, 29 (50%) out of 58 courses were started within the first 3 days of life, 4 (7%) between day three and ten and 25 (43%) after day ten.

### Indications for Antibiotic Courses

**Figure 2:**
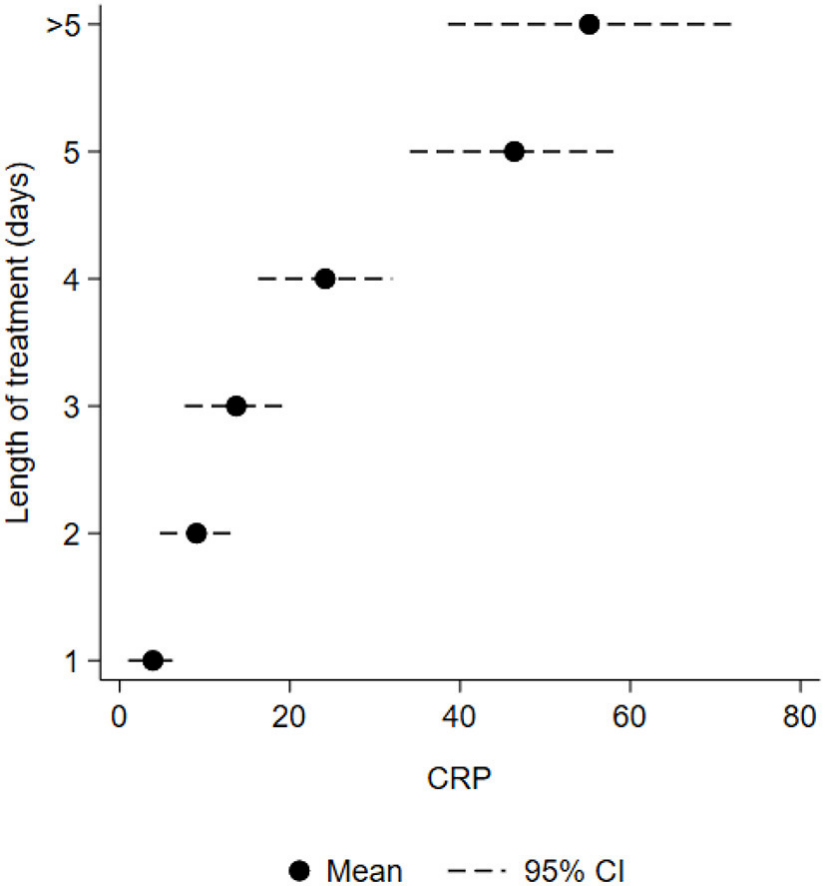
Mean maximum CRP level (*n* = 120) in relation to number of treatment days (GA ≥ 28 weeks) among Norwegian hospitalized neonates.

**Figure 3:**
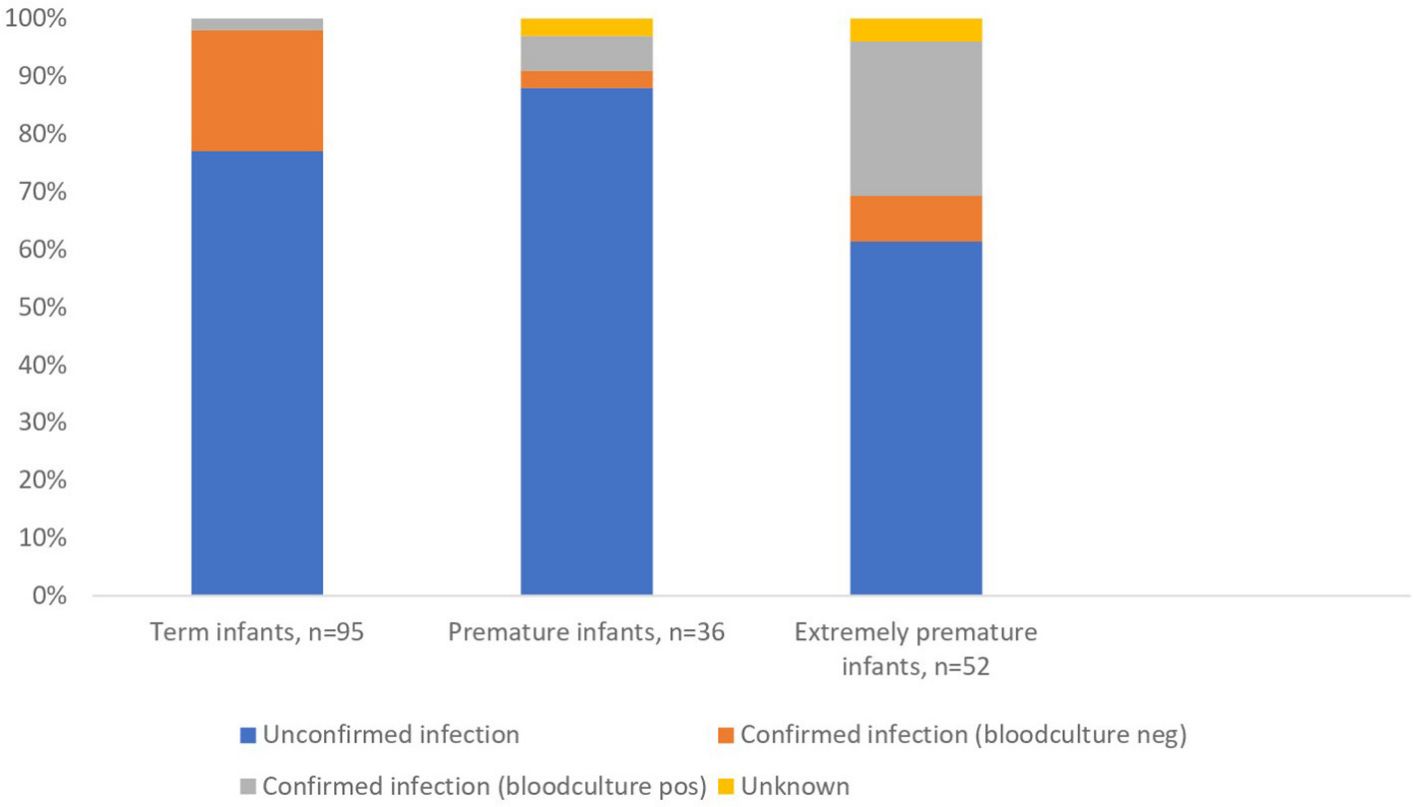
Treatments for early-onset sepsis and late-onset sepsis in two Norwegian neonatal units differentiating between confirmed and unconfirmed infections proportionally.

For term and premature infants, treatment of suspected EOS accounted for 121 (81%) out of 150 antibiotic courses without significant difference between the hospitals (78% at the UH and 84% at the DH, *p* = 0.452). The remaining courses were given for LOS (7%), organ-specific infections without suspected sepsis (7%) and prophylaxis (5%). See Table 2 for detailed information about the various indications. Table 3 shows characteristics in treatments of confirmed and unconfirmed EOS among term and premature infants, and highlights that a high number of treatments were given for unconfirmed EOS. Median treatment duration for unconfirmed EOS was 3 days both for term and premature infants and without significant difference between the hospitals (Table 3). The maximum CRP value (mean) and the confident intervals increased parallel to the number of treatment days (Figure 2). The estimated correlation coefficient was 0.64 (*p* < 0.001). Among extremely premature infants, EOS accounted for 28 (48%) out of 58 antibiotic courses, late-onset sepsis (LOS) for 24 (41%) of the courses and prophylaxis for 6 (10%) of the courses. Mean treatment duration was 4.25 days, 95% CI 3.49–5.01 (EOS + LOS). Figure 3 shows that a much higher proportion of infants received treatment for confirmed infections among extremely premature infants. See Supplemental Digital Content 1 for more detailed characteristics of the extremely premature infants. In total, two patients died during their antibiotic therapy (one at the UH and one at the DH).

**Table 3:** Characteristic in treatment of early-onset sepsis (EOS) in two Norwegian neonatal units, gestational age (GA) ≥ 28 weeks.

	**All**	**University Hospital**	**District Hospital**	***P*-value^a^**
**All**				
EOS treatments, *n*	121	48	73	
Confirmed EOS^b^, *n* (%, 95% CI)	21 (17, 10–24)	11 (23, 11–35)	10 (14, 6–22)	
Unconfirmed EOS, *n* (%, 95% CI)	99 (82, 75–89)	36 (75, 63–87)	63 (86, 78–94)	
Unknown (%)	1 (0.8)	1 (2)	0 (0)	0.172
**Term infants (GA** **≥37 weeks)**				
EOS treatments, *n* (%)	91 (75)	36 (75)	55 (75)	0.966
**Confirmed EOS**				
Treatments, *n* (%, 95% CI)	21 (23, 14–32)	11 (31, 16–46)	10 (18, 8–28)	0.173
Treatment duration, mean (95% CI)	5.95 (5.4–6.5)	6.1 (5.3–6.9)	5.8 (5.3–6.3)	0.586
Maximum CRP, mean (95% CI)	61.1 (52.4–69.8)	61.0 (48.4–73.6)	61.3 (49.5–73.1)	0.975
Bloodcultures obtained, *n* (%)	21 (100)	11 (100)	10 (100)	n/a
Positive bloodcultures, *n* (%, 95% CI)	2^c^ (10, 0–22)	1 (10, 0–26)	1 (10, 0–29)	0.945
Respiratory support, *n* (%)	5 (24)	4 (36)	1 (10)	0.169
**Unconfirmed EOS**				
Treatments, *n* (% 95% CI)	70 (77, 68–86)	25 (69, 54–84)	45 (82, 72–92)	0.173
Treatment duration, mean (95% CI)	3.01 (2.7–3.3)	3.2 (2.4–3.9)	3.0 (2.7–3.3)	0.709
Maximum CRP, mean (95% CI)	17.3 (12.9–21.5)	18.2 (12.0–24.5)	16.8 (11.6–22.9)	0.751
Bloodcultures obtained, *n* (%)	69 (99)	24 (96)	45 (100)	0.357
Respiratory support, *n* (%)	28 (40)	11 (44)	17 (38)	0.613
**Premature infants (28–36 weeks)**				
EOS treatments, *n* (%)	30 (25)	12 (25)	18 (25)	0.966
**Confirmed EOS**				
Treatments, *n* (%)	0 (0)	0 (0)	0 (0)	n/a
**Unconfirmed EOS**				
Treatments, *n* (%, 95% CI)	29 (97, 90–100)	11 (92, 76–100)	18 (100, 81–100)	0.221
Treatment duration, mean (95% CI)	3.03 (2.6–3.5)	3.4 (2.5–4.2)	2.8 (2.4–3.3)	0.313
Maximum CRP, mean (95% CI)	8.6 (4.1–13.1)	5.9 (−0.65–12.45)	10.2 (3.42–17.02)	0.305
Bloodculture obtained, *n* (%)	28 (97)	11 (100)	17 (94)	0.434
Respiratory support, *n* (%)	20 (69)	8 (73)	12 (67)	0.737
**Unknown**				
Treatments, *n* (%)	1 (3.3)	1 (8.3)	0 (0)	0.222

a *Chi square test was used for proportions and Student's t-test for means. For “all treatments,” p-value was based on chi square test for all variables in the section*.

b *Positive blood culture or CRP > 30 and minimum five days of treatment (or death before 5 days). Bloodcultures with Coagulase-negative staphylococci (CoNS) were considered positive if CRP > 10 and minimum 5 days of treatment (or death before 5 days)*.

c *One case of Streptococcus agalacticae (GBS) at the University hospital and one case of Staplylococcus epidermidis at the District hospital*.

### Blood Cultures

The rate of blood cultures obtained before initiation of treatments for sepsis (all GA groups) was 99% (171/173). Among term and premature infants, four (two EOS and two LOS) out of 128 (3.1%, 95% CI 0.0–6.2) treatments for suspected sepsis revealed a positive blood culture, corresponding to 0.8/1,000 live born infants. For EOS, the numbers needed to treat for one positive blood culture was 60. Among extremely premature infants, 14 (2 EOS and 12 LOS) out of 52 (27%, 95% CI 13–35) treatments for sepsis included a positive blood culture (12 at the UH and 2 at the DH), corresponding to 14 out of 38 (37%) of extremely premature infants in the units. Figure 3 clearly illustrates that the proportion of blood culture positive infections was much higher for extremely premature infants.

Overall, the bacteria growing in the cultures were *coagulase negative Staphylococcus* (8), *Streptococcus agalactiae* (5), *Staphylococcus aureus* (3), and *Escherichia coli* (2). Mean treatment duration for infections with *coagulase negative Staphylococcus* was 7.0 days. Five of the treatments involved vancomycin supplemented with one or more of the following antibiotics: aminoglycoside, ampicillin, cloxacillin or ceftazidime. One treatment involved only Cefotaxim and the two last treatments involved an aminoglycoside combined with ampicillin in one case and cloxacillin in the other. Mean treatment duration for infections with *Streptococcus agalactiae* was 9.3 days. Two of the treatments involved mainly benzylpenicillin (partly in combination with an aminoglycoside), two treatments involved an aminoglycoside combined with ampicillin in one case and cloxacillin in the other, and one case involved a combination of vancomycin, ceftazidime, and metronidazole. Mean treatment duration for infections with *Staphlococcus aureus* was 8.3 days. Two of the treatments consisted of cloxacillin monotherapy for more than half of the course and the last case involved an aminoglycoside combined with ampicillin and cloxacillin. For treatment of *Escherichia coli*, one patient was treated with an aminoglycoside combined with ampicillin for 8 days. The other patients, that was treated with an aminoglycoside and ampicillin the first to days and with cefotaxime monotherapy the third day, died during the treatment period. Among the other patients with culture positive sepsis, no fatalities or relapse of infections were registered during the study period.

### Doses

Among term infants treated with antibiotics during the first week of life, significantly higher doses of aminoglycosides were used at the UH compared to the DH (Table 4). Moreover, the number of daily administrations for ampicillin was higher at the DH.

**Table 4:** Doses of aminoglycosides and ampicillin among term born infants first 10 days of life in two Norwegian neonatal units, 2017.

**Antibiotic**	**University Hospital**	**District Hospital**	***P*-value^**a**^**
**AMINOGLYCOSIDE**			
Administrations per day^b^ (*n*), number, mean (95% CI)	39, 0.97 (0,90–1.04)	55, 0.99 (0.97–1.01)	0.912
Dose (mg/kg/day), number, median (95% CI)	39, 5.96 (5.02–6.89)	55, 4.98 (4.82–5.14)	<0.001
**AMPICILLIN**			
Administrations per day^b^^,^ ^c^ (*n*), number, mean (95% CI)	37, 2.00 (n/a)	55, 2.20 (2.09–2.32)	0.002
Dose (mg/kg/day), number, median (95% CI)	37, 100 (98.48–101.52)	55, 100 (93.89–106.11)	0.248

a *Student's t test was used for means and Mood median test for medians.*

b *Number of single doses of antibiotics given within 24 h.*

c *DH: Ten daily doses were administered in three daily administrations and one daily dose was administered in four daily administrations (all were 0–3 days old). UH: All daily doses were administered in two daily administrations.*

*• No switch of daily doses was registered for any of the antibiotics during one single course.*

## Discussion

This study reveals that only 1/5 of treatments for suspected EOS in term and premature infants were confirmed infections. Average treatment length for unconfirmed infections was just above 3 days. No significant differences were observed between the hospitals for characteristics of EOS, but doses and choice of aminoglycosides varied between the hospitals.

A strength of this study is the prospective design, that only a few collectors performed the registrations and the small share of missing data. Other studies have excluded coagulase-negative Staphylococci from epidemiological overviews of positive blood cultures because of the probability of contamination (11, 19). Since we included both the treatment length and the CRP values in our data collection, we could apply the definition from the Norwegian Neonatal Network to decide whether or not to regard these as “true” positive cultures (18). An advantage of using an observational cohort design instead of a point-prevalence survey is the lower risk for casualties like ongoing epidemics, to influence the results. Furthermore, our design gives access to variables that requires continuously observational data for the entire treatment period of an antibiotic course. However, a disadvantage with long period registrations is the challenge and feasibility to include more than just a few hospitals.

An important limitation is the low power of the study to detect clinically relevant differences between the hospitals. We aimed to include extremely premature infants also from the DH, but this could not be done due to unexpected hospital centralization for infants with GA <26 weeks during the study period. Another limitation is the lack of data on maternal risk factors for EOS. We did nor register whether patients were admitted from home, from other hospitals or from the maternity ward; as described in methods we could speculate that some patients at the DH were admitted from home reflected by the significantly higher number of term and premature infants >10 days when initiating their antibiotic course. The data collection was performed by clinicians and nurses working at the respective wards, and most clinicians were aware of the study. This may have affected the data in the manner of more prudent antibiotic use than usual. However, as this possible bias was the same in both hospitals it would not affect the comparison between the hospitals. Our sampling did neither report the name of the clinicians prescribing antibiotics. It is reasonable to assume that prescription habits are difficult to change in a manner that would have a significantly impact on our results, but a minor bias can not be excluded. Finally, different registration periods at the two hospitals may have introduced a bias in relation to the seasonality of certain pathogens. One study concluded that there was no seasonal variation in the prevalence of gram-negative microbes causing LOS (20). Another study showed a prevalence of viral infections of 1% at admitted neonates in a neonatal unit (21). Viral infections are known for their seasonality and may have created an imbalance in the two registrations that should be taken into account as they often lead to antibiotic use in infants. However, both hospitals have strict infection control and isolation routines at their neonatal wards, and our main objective of this study was nor to describe the prevalence of infections.

For term and premature infants, use of BSA was low in both hospitals, but the number of prescriptions was higher at the DH. This can partly be explained by four children at the DH receiving BSA after 10 days of age compared to zero at the UH. Also, three of the seven BSA treatments at the DH was for other indications than sepsis and three of the infants were critically ill in term of receiving respirator treatment. One could speculate whether doctors at the UH have a higher threshold for prescribing BSA than in the DH, but taking the low numbers of BSA treatments into account, this difference is probably not clinically relevant. However, this finding should be controlled in future studies.

The difference in the doses of aminoglycosides in term infants is explained by different local guideline recommendations at the hospitals. We did not register any switch of aminoglycoside doses in term infants during the same course, and one study showed that aminoglycosides safely can be dosed 6 mg/kg every 24 h in term born infants (22). The higher number (mean) of daily administrations with ampicillin at the DH may be explained by the recommendation in a commonly used local Norwegian neonatal supervisor to increase the number of administrations from two up to four per day in severe infections/meningitis (23). From our data, we can not conclude whether there was a difference in severity between the hospitals. More studies focusing on therapeutic drug monitoring of antibiotics in neonates should be conducted in order to optimize dose regimes in the future (24).

The choice of aminoglycoside differed in the UH (gentamicin) and the DH (tobramycin) because of different local guidelines. One study found lower creatinine levels in neonates treated with tobramycin compared to gentamicin, but concluded that the clinical significance of the findings were minimal (25). Tobramycin is the preferred antibiotic to treat infections caused by *Pseudomonas aeruginosa* (26), but this pathogen is rarely detected in the Norwegian infant population (11, 27). As tobramycin is more expensive than gentamicin, the latter can be argued as the aminoglycoside of choice for neonates. By exploring the local hospital guideline at the DH, we could not find any specific reasons (such as local data on microbiological resistance patterns) that would support the use of tobramycin, and from 2018 the DH started to recommend the use of gentamicin as first choice aminoglycoside, partly because of this review of practice.

The rate of antibiotic exposure during the first 3 days of life in term infants is in line with national data from 2009 to 2011 (11), 2.1 and 2.2%, respectively. This indicates no significant change in antibiotic exposure during the last 6 years. However, our rate is low, compared to international literature (28–30). The relation between culture-positive and culture-negative sepsis (term infants, EOS) in our population (1:10) is in the published range (31), while the numbers needed to treat for one positive culture [60] is at the lower side of the literature (11, 32). Our rate of antibiotic exposure for extremely premature infants is in line with data from the USA (33).

We found that 77% of antibiotic courses to term infants for suspected EOS were given to infants without confirmed infections, compared to 54% in the previous national survey (11). In the national survey all antibiotic exposures (including prophylaxis) was included, but only two term infants in our study received prophylactic courses. Also, criteria for culture negative sepsis varied, as the national survey did not include CRP values in the evaluation, and therefore may have overestimated the incidence of confirmed infections. Among premature infants, we observed no confirmed infections in any of the hospitals, indicating a lower threshold for antibiotic therapy. Several studies show that introduction of an algorithm/observational based risk stratification strategy for neonatal sepsis can reduce antibiotic use (30, 34).

Mean treatment duration for unconfirmed EOS (term infants) was shorter than in the national survey from 2009 to 2011, 3 (mean) vs. 4 days (median), respectively. Extremely premature infants in our study were averagely treated 4 days (EOS+LOS). The probability of positive blood cultures beyond 24–48 h is small (35), and studies indicate that treatment safely can be withdrawn after 48 h when clinical suspicion is low (36, 37).

We found that maximum CRP values and 95% CI increased with number of treatment days, indicating that CRP values are regarded as an important factor when deciding treatment length. A recently published study showed that CRP values >30 was uncommon in healthy term infants, supporting the decision of using 30 as cut of level for infection (38). Other biomarkers are also used, but available evidence has not concluded which to prefer (39).

In choice of antibiotics, adherence to local hospital guidelines was high and the use of BSA was low. Previous studies show that several Norwegian hospitals use benzylpenicillin instead of ampicillin in empirical treatments combined with an aminoglycoside (11, 16, 40), and this variation is also present world-wide (41). High prevalence of *Listeria monocytogenes* could justify the use of ampicillin, but according to data from The Norwegian Institute of Public Health, only four cases of listeriosis have been reported among Norwegian children (< 1 year) from 2011 to 2018 (42). Use of ampicillin combined with gentamicin may increase the selection of resistant gram negative bacteria in neonatal units (43). A randomized controlled trial comparing the two regimes found no difference in efficiency or in gut disturbance, but it was underpowered to detect clinical differences (44). Nevertheless, since benzylpenicillin has a narrower antibacterial spectrum probably leading to a lower risk of gut disturbance and resistance, we suggest both study hospitals to consider benzylpenicillin instead of ampicillin in their local guidelines. The commonly used local (but national available) Norwegian neonatal supervisor also recommends benzylpenicillin on behalf of ampicillin. At the DH, this change was performed in their local guidelines during 2018.

The definition of medical prophylaxis in neonates is complicated and not well-established. We introduce a definition combining symptoms and the obtainment of blood culture (there is no need for blood culture if the purpose is to prevent an infection) to rule out prophylaxis. Even though we speculate that blood cultures in some cases are taken as part of an implemented routine, our results show a low use of antibiotic prophylaxis in both hospitals compared to international data (41).

The number of extremely premature infants with a positive blood culture (37% of all extremely premature infants in the units, EOS and LOS), is in line with international reports (45, 46). It confirms the need for new strategies to prevent infections in these vulnerable neonates. However, one study identified that one third of extremely premature infants had low risk of EOS and possibly could avoid exposure to antibiotics (47).

Our results can be used in future antibiotic stewardship programs, including research projects, in Norwegian neonatal departments, for instance by introducing interventions/algorithms to reduce antibiotic exposure and treatment duration. A unified national guideline including clear antibiotic recommendations and dose regimes is desirable. For future surveillances, we have suggested a definition of prophylaxis.

## Conclusion

Based on our study there are no indications of major differences in the pattern of antibiotic use between university and district hospitals in Norway, but term infants at the UH were treated with higher doses of aminoglycosides and fewer daily administrations of ampicillin. Furthermore, gentamicin was the aminoglycoside of choice at the UH, while tobramycin was mainly used at the DH. Even though neonates in Norway receive less antibiotic than in other countries, this study revealed that there is a potential for reduction in both antibiotic exposure and treatment duration for neonates. A systematic risk/observational stratification of sepsis should be considered in both hospitals.

## Data Availability Statement

The raw data supporting the conclusions of this manuscript will be made available by the authors, without undue reservation, to any qualified researcher.

## Ethics Statement

The studies involving human participants were reviewed and approved by Regional Committee for Medical and Health Research Ethics (2017/30/REK Midt). Written informed consent from the participants' legal guardian/next of kin was not required to participate in this study in accordance with the national legislation and the institutional requirements.

## Author Contributions

CT, DB, HB, and BE were involved in the development of the protocol. CT developed the registration form and was responsible for the data collection at Ålesund hospital. AB was responsible for the data collection at Oslo University Hospital, Ullevål. CT and TM did the analyses. CT wrote the first draft. All the authors contributed to the interpretation of the data and revisions of the manuscript and approved the final version of the manuscript.

### Conflict of Interest

The authors declare that the research was conducted in the absence of any commercial or financial relationships that could be construed as a potential conflict of interest.

## Abbreviations

UH: University hospital, DH, District hospital
AMR: Antimicrobial resistance
BSA: Broad-spectrum antibiotics
GA: Gestational age
CNS: Central nervous system.

## References

[1] Goossens H. Antibiotic consumption and link to resistance. Clin Microbiol Infect. (2009) 15(Suppl 3):12–5. 10.1111/j.1469-0691.2009.02725.x 19366364

[2] Roca I Akova M Baquero F Carlet J Cavaleri M Coenen S. The global threat of antimicrobial resistance: science for intervention. N Microbes N Infect. (2015) 6:22–9. 10.1016/j.nmni.2015.02.007 26029375 PMC4446399

[3] NORM/NORM-VET 2017. Usage of Antimicrobial Agents and Occurrence of Antimicrobial Resistance in Norway. Tromsø; Oslo: (2018).

[4] Norwegian Ministry of Health and Care Services. National Strategy against Antibiotic Resistance 2015–2020. Report number: I-1164 E. Oslo (2015).

[5] Korpela K Salonen A Virta LJ Kekkonen RA Forslund K Bork P. Intestinal microbiome is related to lifetime antibiotic use in Finnish pre-school children. Nat Commun. (2016) 7:10410. 10.1038/ncomms10410 26811868 PMC4737757

[6] de Man P Verhoeven BA Verbrugh HA Vos MC van den Anker JN. An antibiotic policy to prevent emergence of resistant bacilli. Lancet. (2000) 355:973–8. 10.1016/S0140-6736(00)90015-1 10768436

[7] Fjalstad JW Esaiassen E Juvet LK van den Anker JN Klingenberg C. Antibiotic therapy in neonates and impact on gut microbiota and antibiotic resistance development: a systematic review. J Antimicrob Chemother. (2017) 3:569–80. 10.1093/jac/dkx426 29182785

[8] Bailey LC Forrest CB Zhang P Richards TM Livshits A DeRusso PA. Association of antibiotics in infancy with early childhood obesity. JAMA Pediatr. (2014) 168:1063–9. 10.1001/jamapediatrics.2014.1539 25265089

[9] Kerr CA Grice DM Tran CD Bauer DC Li D Hendry P. Early life events influence whole-of-life metabolic health via gut microflora and gut permeability. Crit Rev Microbiol. (2015) 41:326–40. 10.3109/1040841X.2013.837863 24645635

[10] Mitre E Susi A Kropp LE Schwartz DJ Gorman GH Nylund CM. Association between use of acid-suppressive medications and antibiotics during infancy and allergic diseases in early childhood. JAMA Pediatr. (2018):e180315. 10.1001/jamapediatrics.2018.0315 29610864 PMC6137535

[11] Fjalstad JW Stensvold HJ Bergseng H Simonsen GS Salvesen B Ronnestad AE. Early-onset sepsis and antibiotic exposure in term infants: a nationwide population-based study in Norway. Pediatr Infect Dis J. (2016) 35:1–6. 10.1097/INF.0000000000000906 26368059

[12] Russell AB Sharland M Heath PT. Improving antibiotic prescribing in neonatal units: time to act. Arch Dis Child Fetal Neonatal Ed. (2012) 97:F141–6. 10.1136/adc.2007.120709 21037285

[13] Cantey JB Baird SD. Ending the culture of culture-negative sepsis in the neonatal ICU. Pediatrics. (2017) 140:e20170044. 10.1542/peds.2017-0044 28928289

[14] Schulman J Dimand RJ Lee HC Duenas GV Bennett MV Gould JB. Neonatal intensive care unit antibiotic use. Pediatrics. (2015) 135:826–33. 10.1542/peds.2014-3409 25896845

[15] Kontou A Sarafidis K Roilides E. Antimicrobial dosing in neonates. Exp Rev Clin Pharmacol. (2017) 10:239–42. 10.1080/17512433.2017.1279968 28067058

[16] Thaulow CM Berild D Eriksen BH Myklebust TA Blix HS. Potential for more rational use of antibiotics in hospitalized children in a country with low resistance - data from eight point prevalence surveys. Pediatr Infect Dis J. (2018) 38:384–9. 10.1097/INF.0000000000002106 30882728

[17] European Centre for Disease Prevention and Control. Point prevalence survey of healthcareassociated infections and antimicrobial use in European acute care hospitals – protocol version 4.3. Stockholm: ECDC (2012).

[18] The Norwegian Medical Association. Forslag til Enhetlige Nasjonale Kriterier for Diagnosekoder i Nyfødtmedisin (In Norwegian). October 2015. Available online at: https://legeforeningen.no/PageFiles/25877/Neonatale%20diagnosekoder%20i%20ICD-10.pdf (accessed October 18, 2018).

[19] Stoll BJ Hansen NI Sanchez PJ Faix RG Poindexter BB Van Meurs KP. Early onset neonatal sepsis: the burden of group B Streptococcal and *E. coli* disease continues. Pediatrics. (2011) 127:817–26. 10.1542/peds.2010-2217 21518717 PMC3081183

[20] Hammoud MS Al-Taiar A Al-Abdi SY Bozaid H Khan A AlMuhairi LM. Late-onset neonatal sepsis in Arab states in the Gulf region: two-year prospective study. Int J Infect Dis. (2017) 55:125–30. 10.1016/j.ijid.2017.01.006 28088587

[21] Verboon-Maciolek MA Krediet TG Gerards LJ Fleer A van Loon TM. Clinical and epidemiologic characteristics of viral infections in a neonatal intensive care unit during a 12-year period. Pediatr Infect Dis J. (2005) 24:901–4. 10.1097/01.inf.0000180471.03702.7f 16220089

[22] Fjalstad JW Laukli E van den Anker JN Klingenberg C. High-dose gentamicin in newborn infants: is it safe? Eur J Pediatr. (2013) 173:489–95. 10.1007/s00431-013-2194-1 24233331

[23] Barne-og ungdomsavdelingen Universitetssykehuseti Nord Norge. Metodebok i Nyfødtmedisin (in Norwegian). 4th ed. Tromsø (2012).

[24] van Donge T Bielicki JA van den Anker J Pfister M. Key Components for antibiotic dose optimization of sepsis in neonates and infants. Front Pediatr. (2018) 6:325. 10.3389/fped.2018.00325 30420947 PMC6215831

[25] Itsarayoungyuen S Riff L Schauf V Hamilton L. Tobramycin versus gentamicin for the neonate. Pediatr Res. (1981) 15:496. 10.1203/00006450-198104001-00346

[26] Brogden R Pinder R Sawyer R Speight MT Avery SG. Tobramycin: a review of its antibacterial and pharmacokinetic properties and therapeutic use. Drugs. (1976) 12:166–200. 10.2165/00003495-197612030-00002 789045

[27] Ronnestad A Abrahamsen TG Medbo S Reigstad H Lossius K Kaaresen PI. Late-onset septicemia in a Norwegian national cohort of extremely premature infants receiving very early full human milk feeding. Pediatrics. (2005) 115:e269–76. 10.1542/peds.2004-1833 15687416

[28] Achten NB Dorigo-Zetsma JW van der Linden PD van Brakel M Plotz FB. Sepsis calculator implementation reduces empiric antibiotics for suspected early-onset sepsis. Eur J Pediatr. (2018) 177:741–6. 10.1007/s00431-018-3113-2 29455368

[29] Escobar GJ Puopolo KM Wi S Turk BJ Kuzniewicz MW Walsh EM. Stratification of risk of early-onset sepsis in newborns ≥ 34 weeks' gestation. Pediatrics. (2014) 133:30–6. 10.1542/peds.2013-1689 24366992 PMC4079292

[30] Kuzniewicz MW Puopolo KM Fischer A Walsh EM Li S Newman TB. A quantitative, risk-based approach to the management of neonatal early-onset sepsis. JAMA Pediatr. (2017) 171:365–71. 10.1001/jamapediatrics.2016.4678 28241253

[31] Klingenberg C Kornelisse RF Buonocore G Maier RF Stocker M. Culture-negative early-onset neonatal sepsis - at the crossroad between efficient sepsis care and antimicrobial stewardship. Front Pediatr. (2018) 6:285. 10.3389/fped.2018.00285 30356671 PMC6189301

[32] Stocker M van Herk W El Helou S Dutta S Fontana MS Schuerman F. Procalcitonin-guided decision making for duration of antibiotic therapy in neonates with suspected early-onset sepsis: a multicentre, randomised controlled trial (NeoPIns). Lancet. (2017) 390:871–81. 10.1016/S0140-6736(17)31444-7 28711318

[33] Flannery DD Ross RK Mukhopadhyay S Tribble AC Puopolo KM Gerber JS. Temporal trends and center variation in early antibiotic use among premature infants. JAMA Network Open. (2018) 1:e180164. 10.1001/jamanetworkopen.2018.0164 30646054 PMC6324528

[34] van Herk W Stocker M van Rossum AM. Recognising early onset neonatal sepsis: an essential step in appropriate antimicrobial use. J Infect. (2016) 72(Suppl):S77–82. 10.1016/j.jinf.2016.04.026 27222092

[35] Durrani N Rochow N Alghamdi J Pelc A Fusch C Dutta S. Minimum duration of antibiotic treatment based on blood culture in ruled out neonatal sepsis. Pediatr Infect Dis J. (2018) 38:528–32. 10.1097/INF.0000000000002182 30169482

[36] Astorga MC Piscitello KJ Menda N Ebert AM Ebert SC Porte MA. Antibiotic stewardship in the neonatal intensive care unit: effects of an automatic 48-hour antibiotic stop order on antibiotic use. J Pediatric Infect Dis Soc. (2018) 8:310–6. 10.1093/jpids/piy043 29846666

[37] Cantey JB Wozniak PS Pruszynski JE Sanchez PJ. Reducing unnecessary antibiotic use in the neonatal intensive care unit (SCOUT): a prospective interrupted time-series study. Lancet Infect Dis. (2016) 16:1178–84. 10.1016/S1473-3099(16)30205-5 27452782

[38] Mjelle AB Guthe HJT Reigstad H Bjorke-Monsen AL Markestad T. Serum concentrations of C-reactive protein in healthy term-born Norwegian infants 48–72 hours after birth. Acta Paediatr. (2018) 108:626–9. 10.1111/apa.14578 30222898

[39] Sharma D Farahbakhsh N Shastri S Sharma P. Biomarkers for diagnosis of neonatal sepsis: a literature review. J Matern Fetal Neonatal Med. (2018) 31:1646–59. 10.1080/14767058.2017.1322060 28427289

[40] Drageset M Fjalstad JW Mortensen S Klingenberg C. Management of early-onset neonatal sepsis differs in the north and south of Scandinavia. Acta Paediatr. (2017) 106:375–81. 10.1111/apa.13698 27935180

[41] Versporten A Bielicki J Drapier N Sharland M Goossens H group Ap. The worldwide antibiotic resistance and prescribing in european children (ARPEC) point prevalence survey: developing hospital-quality indicators of antibiotic prescribing for children. J Antimicrob Chemother. (2016) 71:1106–17. 10.1093/jac/dkv418 26747104

[42] Blystad H. Smittevernveiledern. Norwegian Institue of Public Health (In Norwegian). Available online at: https://www.fhi.no/nettpub/smittevernveilederen/sykdommer-a-a/listeriose—veileder-for-helsepers/#forekomst-i-norge (accessed September 25, 2019).

[43] Crivaro V Bagattini M Salza MF Raimondi F Rossano F Triassi M. Risk factors for extended-spectrum beta-lactamase-producing Serratia marcescens and Klebsiella pneumoniae acquisition in a neonatal intensive care unit. J Hosp Infect. (2007) 67:135–41. 10.1016/j.jhin.2007.07.026 17884248

[44] Metsvaht T Ilmoja ML Parm U Maipuu L Merila M Lutsar I. Comparison of ampicillin plus gentamicin vs. penicillin plus gentamicin in empiric treatment of neonates at risk of early onset sepsis. Acta Paediatr. (2010) 99:665–72. 10.1111/j.1651-2227.2010.01687.x 20096030

[45] Stoll BJ Hansen NI Bell EF Shankaran S Laptook AR Walsh MC. Neonatal outcomes of extremely preterm infants from the NICHD Neonatal Research Network. Pediatrics. (2010) 126:443–56. 10.1542/peds.2009-2959 20732945 PMC2982806

[46] Neill S Haithcock S Smith PB Goldberg R Bidegain M Tanaka D. Sustained reduction in bloodstream infections in infants at a large tertiary care neonatal intensive care unit. Adv Neonatal Care. (2016) 16:52–9. 10.1097/ANC.0000000000000164 25915573 PMC4619157

[47] Puopolo KM Mukhopadhyay S Hansen NI Cotten CM Stoll BJ Sanchez PJ. Identification of extremely premature infants at low risk for early-onset sepsis. Pediatrics. (2017) 140:e20170925. 10.1542/peds.2017-0925 28982710 PMC5654397

