# Corrigendum: Can We Optimize Antibiotic Use in Norwegian Neonates? A Prospective Comparison Between a University Hospital and a District Hospital

**DOI:** 10.3389/fped.2022.862484

**Published:** 2022-03-10

**Authors:** Christian Magnus Thaulow, Dag Berild, Hege Salvesen Blix, Anne Karin Brigtsen, Tor Åge Myklebust, Beate Horsberg Eriksen

**Affiliations:** ^1^Clinical Institute II, University of Bergen, Bergen, Norway; ^2^Department of Pediatrics, Haukeland University Hospital, Bergen, Norway; ^3^Institute of Clinical Medicine, University of Oslo, Oslo, Norway; ^4^Institute of Pharmacology, University of Oslo, Oslo, Norway; ^5^Department of Infectious Diseases, Oslo University Hospital, Oslo, Norway; ^6^Department of Drug Statistics, Norwegian Institute of Public Health, Oslo, Norway; ^7^Department of Pediatrics, Oslo University Hospital, Oslo, Norway; ^8^Department of Research and Innovation, Møre and Romsdal Hospital Trust, Alesund, Norway; ^9^Department of Pediatrics, Møre and Romsdal Hospital Trust, Oslo, Norway

**Keywords:** neonatal antibiotic use, antimicrobial resistance, pediatric antibiotic stewardship, antibiotic doses, antibiotic prescriptions

In the original article, in Figure 2, the lines related to each mean value showed ±2 standard deviations from the 95% confidence intervals. The corrected Figure 2 appears below.

**Figure 2:**
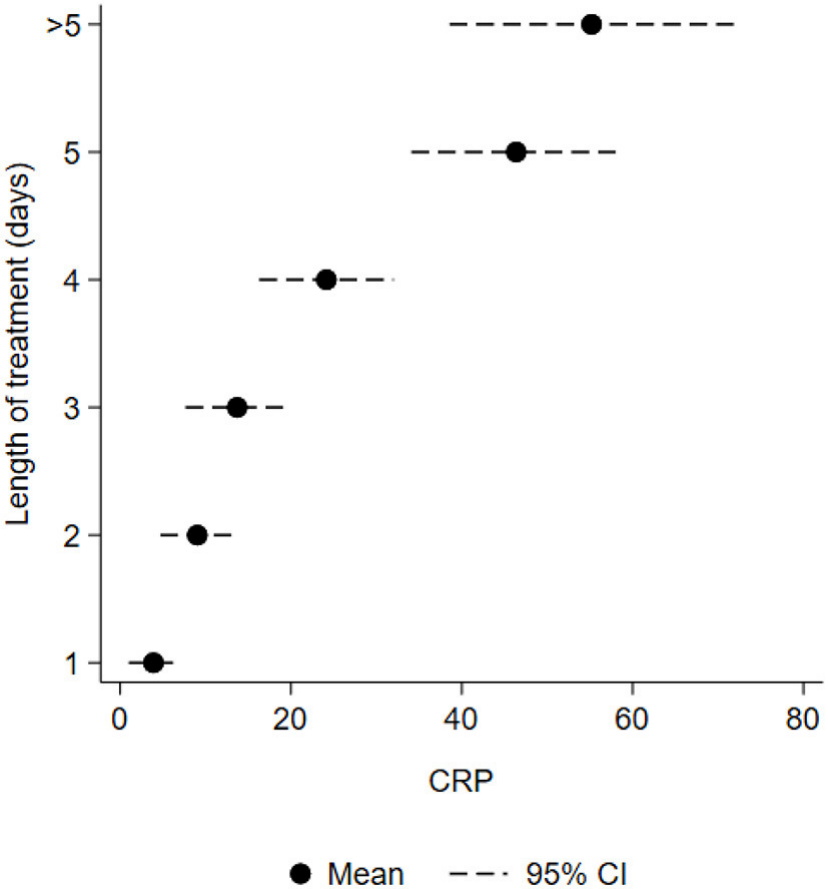
Mean maximum CRP level (*n* = 120) in relation to number of treatment days (GA ≥ 28 weeks) among Norwegian hospitalized neonates.

In the original article, there was a mistake in Table 3, 95% confidence intervals presented on the first line in the section describing treatments for unconfirmed EOS in premature infants were not correct, 91–103 should have been 90–100, 77–107 should have been 76–100 and 100 should have been 81–100. Moreover, 95% confidence intervals presented in the fifth line are not correct; 0–14 should be 0–22 for all, 0–14 should be 0–26 for the University Hospital and 0–14 should be 0–29 for the District Hospital. The corrected Table 3 appears below.

**Table 3:** Characteristic in treatment of early-onset sepsis (EOS) in two Norwegian neonatal units, gestational age (GA) ≥ 28 weeks.

	**All**	**University Hospital**	**District Hospital**	***P*-value^a^**
**All**				
EOS treatments, *n*	121	48	73	
Confirmed EOS^b^, *n* (%, 95% CI)	21 (17, 10–24)	11 (23, 11–35)	10 (14, 6–22)	
Unconfirmed EOS, *n* (%, 95% CI)	99 (82, 75–89)	36 (75, 63–87)	63 (86, 78–94)	
Unknown (%)	1 (0.8)	1 (2)	0 (0)	0.172
**Term infants (GA** **≥37 weeks)**				
EOS treatments, *n* (%)	91 (75)	36 (75)	55 (75)	0.966
**Confirmed EOS**				
Treatments, *n* (%, 95% CI)	21 (23, 14–32)	11 (31, 16–46)	10 (18, 8–28)	0.173
Treatment duration, mean (95% CI)	5.95 (5.4–6.5)	6.1 (5.3–6.9)	5.8 (5.3–6.3)	0.586
Maximum CRP, mean (95% CI)	61.1 (52.4–69.8)	61.0 (48.4–73.6)	61.3 (49.5–73.1)	0.975
Bloodcultures obtained, *n* (%)	21 (100)	11 (100)	10 (100)	n/a
Positive bloodcultures, *n* (%, 95% CI)	2^c^ (10, 0–22)	1 (10, 0–26)	1 (10, 0–29)	0.945
Respiratory support, *n* (%)	5 (24)	4 (36)	1 (10)	0.169
**Unconfirmed EOS**				
Treatments, *n* (% 95% CI)	70 (77, 68–86)	25 (69, 54–84)	45 (82, 72–92)	0.173
Treatment duration, mean (95% CI)	3.01 (2.7–3.3)	3.2 (2.4–3.9)	3.0 (2.7–3.3)	0.709
Maximum CRP, mean (95% CI)	17.3 (12.9–21.5)	18.2 (12.0–24.5)	16.8 (11.6–22.9)	0.751
Bloodcultures obtained, *n* (%)	69 (99)	24 (96)	45 (100)	0.357
Respiratory support, *n* (%)	28 (40)	11 (44)	17 (38)	0.613
**Premature infants (28–36 weeks)**				
EOS treatments, *n* (%)	30 (25)	12 (25)	18 (25)	0.966
**Confirmed EOS**				
Treatments, *n* (%)	0 (0)	0 (0)	0 (0)	n/a
**Unconfirmed EOS**				
Treatments, *n* (%, 95% CI)	29 (97, 90–100)	11 (92, 76–100)	18 (100, 81–100)	0.221
Treatment duration, mean (95% CI)	3.03 (2.6–3.5)	3.4 (2.5–4.2)	2.8 (2.4–3.3)	0.313
Maximum CRP, mean (95% CI)	8.6 (4.1–13.1)	5.9 (−0.65–12.45)	10.2 (3.42–17.02)	0.305
Bloodculture obtained, *n* (%)	28 (97)	11 (100)	17 (94)	0.434
Respiratory support, *n* (%)	20 (69)	8 (73)	12 (67)	0.737
**Unknown**				
Treatments, *n* (%)	1 (3.3)	1 (8.3)	0 (0)	0.222

a *Chi square test was used for proportions and Student's t-test for means. For “all treatments,” p-value was based on chi square test for all variables in the section*.

b *Positive blood culture or CRP > 30 and minimum five days of treatment (or death before 5 days). Bloodcultures with Coagulase-negative staphylococci (CoNS) were considered positive if CRP > 10 and minimum 5 days of treatment (or death before 5 days)*.

c *One case of Streptococcus agalacticae (GBS) at the University hospital and one case of Staplylococcus epidermidis at the District hospital*.

The authors apologize for these errors and state that this does not change the scientific conclusions of the article in any way. The original article has been updated.

## Publisher's Note

All claims expressed in this article are solely those of the authors and do not necessarily represent those of their affiliated organizations, or those of the publisher, the editors and the reviewers. Any product that may be evaluated in this article, or claim that may be made by its manufacturer, is not guaranteed or endorsed by the publisher.

