# Supplementary material for: Can We Optimize Antibiotic Use in Norwegian Neonates? A Prospective Comparison Between a University Hospital and a District Hospital
Source: Front Pediatr. 2019 Oct 24;7:440. doi: 10.3389/fped.2019.00440 (PMC6821648; doi:10.3389/fped.2019.00440)
Supplement: Supplementary file 1 [file Table_1.DOCX]

SDC 1) Characteristic in treatment of neonatal sepsis in extremely premature infants (GA < 28), data from two Norwegian hospitals, 2017

| **Indications** | **Total** |
| --- | --- |
| **All indications** |  |
| Courses, n | 58 |
| Treatments for EOS, n (%) | 28 (48) |
| Treatments for LOS, n (%) | 24 (41) |
| Prophylaxis^1^, n (%) | 6 (10) |
|  |  |
| **Sepsis (EOS and LOS)** |  |
| ***Confirmed sepsis^2,3^*** |  |
| Treatments, n (% of all sepsis treatments) | 18 (35) |
| Treatment length, mean (95% CI) | 7.75 (6.72-8.78) |
| Maximum CRP, mean (95% CI) | 79.8 (60.5-99.1) |
| Bloodcultures obtained, n (%) | 18 (100) |
| Positive bloodcultures^3^, n (%, 95% CI) | 14 (78, 52-94) |
| ***Unconfirmed sepsis*** |  |
| Treatments n (% of all sepsis treatments) | 32(62) |
| Treatment length, mean (95% CI) | 4.25 (3.49-5.01) |
| Maximum CRP, mean (95% CI) | 5.36 (2.80-7.92) |
| Bloodcultures obtained, n (%) | 32 (100) |
| ***Unknown*** |  |
| Treatments, n (%) | 2 (4) |

1. ^Surgical prophylaxis (1), risk for respiratory tract infection (1), unknown (4)^
2. ^Positive blood culture or CRP > 30 and minimum five days of treatment (or death before five days). Bloodcultures with Coagulase-negative staphylococci (CoNS) were considered positive if CRP > 10 and minimum five days of treatment (or death before five days)^
3. ^One case of fatality^
4. ^Coagulase negative Staphylococcus (7), Streptococcus agalacticae (4), Escherichia coli (2), Staphylococcus aureus (1)^

- ^EOS (Early-onset sepsis), LOS (Late-onset sepsis)^
